# A Novel Homozygous Founder Variant of *RTN4IP1* in Two Consanguineous Saudi Families

**DOI:** 10.3390/cells11193154

**Published:** 2022-10-07

**Authors:** Mazhor Aldosary, Maysoon Alsagob, Hanan AlQudairy, Ana C. González-Álvarez, Stefan T. Arold, Mohammad Anas Dababo, Omar A. Alharbi, Rawan Almass, AlBandary AlBakheet, Dalia AlSarar, Alya Qari, Mysoon M. Al-Ansari, Monika Oláhová, Saif A. Al-Shahrani, Moeenaldeen AlSayed, Dilek Colak, Robert W. Taylor, Mohammed AlOwain, Namik Kaya

**Affiliations:** 1 Translational Genomics Department, Center for Genomic Medicine, King Faisal Specialist Hospital and Research Centre (KFSHRC), P.O. Box 3354, Riyadh 11211, Saudi Arabia; 2 Center of Excellence for Biomedicine, Joint Centers for Excellence Program, King Abdulaziz City for Science and Technology (KACST), Riyadh 11442, Saudi Arabia; 3 Bioscience Program, Biological and Environmental Science and Engineering Division, King Abdullah University of Science and Technology (KAUST), Thuwal 23955-6900, Saudi Arabia; 4 Computational Biology Research Center, King Abdullah University of Science and Technology, Thuwal 23955-6900, Saudi Arabia; 5 Centre de Biologie Structurale (CBS), INSERM, CNRS, Université de Montpellier, F-34090 Montpellier, France; 6 Department of Pathology and Laboratory Medicine, King Faisal Specialist Hospital and Research Centre (KFSHRC), P.O. Box 3354, Riyadh 11211, Saudi Arabia; 7 Radiology Department, King Faisal Specialist Hospital and Research Centre (KFSHRC), P.O. Box 3354, Riyadh 11211, Saudi Arabia; 8 Department of Medical Genomics, Center for Genomic Medicine, King Faisal Specialist Hospital and Research Centre (KFSHRC), P.O. Box 3354, Riyadh 11211, Saudi Arabia; 9 Department of Botany and Microbiology, College of Science, King Saud University, Riyadh 11451, Saudi Arabia; 10 Department of Molecular Oncology, King Faisal Specialist Hospital and Research Centre (KFSHRC), P.O. Box 3354, Riyadh 11211, Saudi Arabia; 11 Welcome Centre for Mitochondrial Research, Translational and Clinical Research Institute, Faculty of Medical Sciences, Newcastle University, Newcastle upon Tyne NE2 4HH, UK; 12 College of Medicine, Alfaisal University, P.O. Box 50927, Riyadh 11533, Saudi Arabia; 13 NHS Highly Specialized Service for Rare Mitochondrial Disorders, Newcastle upon Tyne Hospitals NHS Foundation Trust, Newcastle upon Tyne NE1 4LP, UK

**Keywords:** *RTN4IP1*, founder variant, missense, age of variant, encephalopathy, optic atrophy, in silico pathogenicity prediction, structural modeling

## Abstract

The genetic architecture of mitochondrial disease continues to expand and currently exceeds more than 350 disease-causing genes. Bi-allelic variants in *RTN4IP1*, also known as Optic Atrophy-10 (OPA10), lead to early-onset recessive optic neuropathy, atrophy, and encephalopathy in the afflicted patients. The gene is known to encode a mitochondrial ubiquinol oxidoreductase that interacts with reticulon 4 and is thought to be a mitochondrial antioxidant NADPH oxidoreductase. Here, we describe two unrelated consanguineous families from the northern region of Saudi Arabia harboring a missense variant (*RTN4IP1*:NM_032730.5; c.475G<T, p.Val159Phe) in the gene. Clinically affected individuals presented with intellectual disability, encephalopathy, ataxia, optic atrophy, and seizures. Based on whole exome sequencing and confirmatory Sanger sequencing, the variant was fully segregated with the phenotype in the families, absent among large ethnically matching controls as well as numerous in-house exomes, and predicted to be pathogenic by different in silico classifiers. Structural modeling and immunoblot analyses strongly indicated this variant to be pathogenic. Since the families belong to one of the tribal inhabitants of Saudi Arabia, we postulate that the variant is likely to be a founder. We provide the estimated age of the variant and present data confirming the disease-causality of this founder variant.

## 1. Introduction

Recent progress in massively parallel sequencing technologies enables rapid and accurate detection of DNA variants. This methodology has already helped to identify the genetic basis of many previously unresolved cases with known and novel hereditary diseases, including mitochondrial disorders [1]. Hereditary optic neuropathies (HON) are a group of disorders affecting the optic nerve and can be transmitted either maternally or demonstrating autosomal or X-linked patterns of inheritance [2]. Such diseases are usually characterized by the degeneration of retinal ganglion cells (RGC), leading to optic nerve atrophy and the impairment of central vision and having mutual underlying pathophysiology of mitochondrial dysfunction [3,4]. 

One of the recently described nuclear genes leading to mitochondrial presentations of HON is *RTN4IP1* (Reticulon 4-Interacting Protein1, MIM: 610502) [3]. RTN4IP1 was first characterized over two decades ago through the search of an adult human brain cDNA library [5]. It was shown as a novel mitochondrial protein interacting with Nogo, a long-sought factor inhibiting the growth of regenerating nerve axons [6,7,8], 

The 396 amino-acid RTN4IP1 has an N-terminal mitochondrial signaling sequence and consists of two domains: an alcohol dehydrogenase (ADH-N) GroES-like domain (Pro71-His147) and zinc-binding dehydrogenase domain (ADH-zinc) (residues Leu247-Ile393). Based on the findings from a yeast-two hybrid system, RTN4IP1 interacts with two subunits of the mitochondrial complex III—Ubiquinol:cytochrome *c* Reductase Core Proteins 1 and 2 (UQCRC1 and UQCRC2, respectively) [5]. A recent study revealed that RTN4IP1 has a function in coenzyme Q (CoQ) biosynthesis and is a mitochondrial NADPH oxidoreductase with antioxidant activity and with structural homology to bacterial quinone oxidoreductase. Using Rtn4ip1-knockout C2C12 cells, the same study showed reduced oxygen consumption rates, ATP production, as well as CoQ9 levels revealing that RTN4IP1 is required for oxidative phosphorylation in mouse muscle [9]. 

Biallelic genetic variants in *RTN4IP1* have been shown to lead to defects in mitochondrial complex I and IV activities in patient-derived fibroblasts; however, the oxygen consumption levels in these patient cells were normal [3]. Another study reported decreased and isolated complex I activity in patient muscles without evidence of mitochondrial complex IV deficiency [10]. The deficiency of RTN4IP1 leads to degeneration of the optic nerve [11]. Pathogenic *RTN4IP1* variants cause numerous clinical features affecting the eyes, including reduced visual acuity, photophobia, and nystagmus. Additionally, in some cases, pathogenic *RTN4IP1* variants cause color vision impairment, optic disc pallor, central scotoma, decreased visual field sensitivity, reduced thickness of the retinal nerve fiber layer, and reduced or absent visual evoked potentials [3,10]. Moreover, *RTN4IP1* mutations may also result in extraocular manifestations appearing as mitochondrial encephalopathies, such as mild ataxia, intellectual disability, and rare generalized seizures [4,10,12,13,14]. Visual dysfunction and abnormalities start in early childhood [3,10,11,14]. 

Here, we present additional cases from two unrelated consanguineous families who harbor a novel homozygous variant in the *RTN4IP1* gene. Through genetic analysis coupled with structural modeling, in silico pathogenicity investigation, and Western blot analysis, we show that the variant is highly likely to be pathogenic, leading to the clinical features observed in our patients. Moreover, we hypothesize that the variant is likely to be a founder, and thus we provide its estimated age. 

## 2. Materials and Methods

### 2.1. Patient Recruitment and Sample Collection

Two consanguineous Saudi families, of which a total of six individuals exhibited mitochondrial encephalopathy, seizures, ataxia, optic atrophy, and mental retardation symptoms, were recruited under institutional review board protocols at Research Center at King Faisal Specialist Hospital, Riyadh, KSA (KFSHRC, IRB-approved protocols, RAC# 2120022). Venous blood samples (5–10 mL) were collected in EDTA tubes from each patient as well as the rest of the family members. Written informed consent was obtained. 

### 2.2. DNA Isolation, PCR and Sanger Sequencing 

The DNA was extracted from the collected blood samples using a PureGene DNA Purification Kit (Gentra Systems, Inc. Minneapolis, MN, USA). The DNA quality and quantity were examined on 1–2% agarose gel and then analyzed by NanoDrop^®^ ND-1000 (NanoDrop Inc., Wilmington, DE, USA). Exonic primers inclusive of intron–exon boundaries were designed using the Primer 3 web tool. The tested primers were run on the DNA samples of the index case as well as family members and control samples using standard PCR protocols. The PCR products were sequenced using standard Sanger sequencing protocols. The results were aligned with a reference sequence, blasted on NCBI webtool, and analyzed using ChromasPro (Technelysium Pty Ltd., South Brisbane, Australia) and SeqMan software (DNASTAR Inc., Madison, WI, USA) from DNASTAR.

### 2.3. Genome-Wide SNP Genotyping and Autozygosity Mapping

Genome-wide SNP genotyping was performed using custom axiom arrays (GeneChip Human Genome-wide SNP Axiom Arrays, Affymetrix Inc., Santa Cruz, CA, USA) according to the manufacturer’s protocols and guidelines (Affymetrix Inc.). Resulted genotypes and quality control of the chip runs were performed using Affymetrix’s Axiom Analysis Suite v2.0. The SNP genotypes were transferred to AutoSNPa software [15], and the genome-wide loss of heterozygosity blocks was detected using the default settings of the software. The shared blocks among the patients were compared to those of healthy individuals in the families. 

### 2.4. Next Generation Sequencing Using Illumina Platform 

Whole-exome sequencing (WES) was performed as previously described [1,16]. Briefly, the samples were prepared and enriched using Illumina kits according to the manufacturer’s standard protocol (Illumina, San Diego, CA, USA), and the library concentrations were checked using Agilent’s QPCR NGS Library Quantification Kit (G4880A) (Agilent Inc., Santa Clara, CA, USA). The samples were pooled (each sample at a final concentration of 10 nM) before the sequencing was carried out. WES was performed on an Illumina platform (HiSeq2000) using TruSeq chemistry. 

### 2.5. Next Generation Sequencing Using Ion Torrent Platform

The samples were processed and sequenced as previously described [16,17]. Briefly, the samples were barcoded and sequenced with Ion PI Chips on an IonTorrent Proton platform. The sample preparation, barcoding, and chip handling were conducted according to the manufacturer’s guidelines and user guides. 

### 2.6. Variant Analysis and Bioinformatics Analysis 

Variant filtering and pathogenicity analyses were evaluated according to standard protocols and up-to-date pipelines applied at the King Faisal Specialist Hospital and Research Center as published before [18,19,20]. Briefly, the variants were filtered using different features such as minor allele frequency (MAF, being <1% in control databases), being in known disease-causing genes, protein loss of function, pathogenic or likely pathogenic according to ACMG (American College of Medical Genetics and Genomics) guidelines [21], and homozygosity. Several bioinformatics prediction tools (SIFT [22], Polyphen2 [23], MutationTaster [24,25], ConDel [26], SNPdryad [27]) were used to predict the pathogenicity of the variant. Next, several publicly available resources were also searched for the presence of the variant and its novelty (listed under web sources). Amino acid sequences from various species were downloaded from the Ensembl database and aligned using ClustalW software. The sequences of 30 amino acids surrounding the variant site were visualized using Jalview and Protean (DNASTAR, Wisconsin, MD, USA). The structure of human RTN4IP1 (residues 45–396) in complex with NADPH was obtained from the PDB (accession number 2VN8). The variant was introduced in silico, and its effect was evaluated using the Pymol program (pymol.org, accessed on 21 February 2022). 

### 2.7. Cell Passaging and Harvesting 

Age-matched, primary skin fibroblasts from the controls and the *RTN4IP* index patient (F1-II-2, Patient 1, Family 1) were grown in high glucose Dulbecco’s modified Eagle’s medium (DMEM) (Sigma-Aldrich, St. Louis, MO, USA) containing 10% fetal calf serum, 1× non-essential amino acids, 50 U/mL penicillin, 50 μg/mL streptomycin, and 50 μg/mL uridine at 37 °C and 5% CO_2_ in a humidified incubator. For harvesting, both the medium and trypsin were pre-warmed at 37 °C in the incubator prior to use. The cells were washed with 1xPBS. Depending on the size of the flask, a sufficient amount of trypsin was added to cover the surface of the flask, which was then incubated at 37 °C for 1–2 min to dislodge the cells from the flask. A complete DMEM growth medium was added to prevent further proteolysis by quenching the trypsin and to keep the cells in suspension. To harvest the cells, they were allowed to reach 90–100% confluency, washed with 1× PBS followed by incubation in a sufficient amount of trypsin, and briefly incubated at 37 °C. The cells were washed again with 1× PBS and then lysed by complete lysis (EDTA-Free) buffer (Roche, Sigma Aldrich) according to manufacturers’ protocols.

### 2.8. Immunoblotting

The total cell lysates were obtained from cultured fibroblasts for both the patients and controls. Twenty µg/µL of protein and 5 µL of sample buffer containing 2% β-Mercaptoethanol were mixed. Lysis buffer was added to a final volume of 10 µL. In addition, 5 µL of sample buffer was added to 5 µL of Kaleidoscope prestained standard. Twenty µg/µL protein samples were prepared using standard methods and were separated by 12% SDS-PAGE and transferred to a 0.45 mm PVDF membrane (MilliporeSigma, Burlington, MA, USA). Protein transfer was carried out using a PVDF membrane (Thermo Fisher Scientific, Waltham, MA, USA) subsequently blocked in 5 % skimmed milk in Tris Buffered-Saline (TBS) Tween 20 (TBST) prior to immunodecoration with a rabbit polyclonal anti-RTN4IP1 Ab (1:1000 Abcam ab155304) and rabbit β-actin Ab (1:10,000 Abcam Cat#ab8227). Proteins were detected using an enhanced chemiluminescence substrate (Thermo Fisher Scientific).

## 3. Results

### 3.1. Clinical Evaluations

#### 3.1.1. Patient 1 (Family 1)

Patient 1 (F1-II-2) in Family 1 is a 26-year-old female, a twin sister of a healthy fraternal twin. She is the index patient. She was born following a normal pregnancy and delivery without complications with a birth weight of 3 kg. The parents are consanguineous, and her younger brother (Patient 3, F1-II-4) and older sister (Patient 2, F1-II-1) have the same condition. She was able to roll over and sit at the appropriate age, but walking was markedly delayed and unbalanced. She had a speech delay, but she attended special classes and was able to write, speak, and read at a primitive level. Abnormal eye movements were noted since birth. Eye examination revealed poorly reactive pupils to light with bilateral nystagmus and severe optic atrophy. At the age of 12 years, an examination showed dysmophic facial features (bulbous nose, curved relatively small ears, hypertelorism, synopsis, large upper lip, and short philtrum), and skin eczema on the limbs since the age of 3 months. She used to have a seizure disorder, which was under good control. Following 2 years of a seizure-free period, she was weaned off medication. However, she relapsed, and seizures restarted in the form of generalized tonic–clonic seizures, with no signs of myoclonic epilepsy and no atonic spells. An EGG was conducted at the age of 16 years old and was severely abnormal, with the presence of the focal area of cortical neuro-irritability in the bioccipital region, indicating a predisposition for epileptic seizures in the form of complex partial and generalized seizures. Tandem MS for acylcarnitine profile, amino acids, and serum biotinidase were normal. Urine organic acid was unremarkable.

#### 3.1.2. Patient 2 (Family 1)

Patient 2 (F1-II-1) in Family 1 is the elder sister of patient 1 (F1-II-2) (only limited clinical data available). She has nystagmus with ataxia and optic atrophy with unsteadiness. Neurodevelopmentally, she was noted to have significant cognitive problems, speech delay, and severe to moderate psychomotor retardation. At the age of 14 years old, she lost the ability to walk. She had frequent episodes of seizures precipitated by fever and head trauma. An EGG was performed at the age of 15 years old, and it showed multiregional epileptic activity seen from the midline and central temporal region, and frequent high amplitude occipital spikes were seen.

#### 3.1.3. Patient 3 (Family 1)

**Figure 1:**
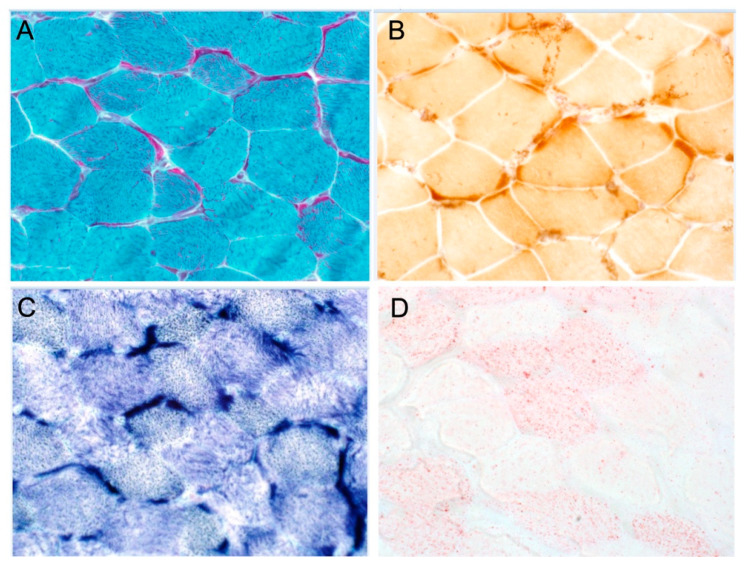
Histochemical and immunohistochemical analyses of muscle biopsy. (**A**) Gomori trichrome staining shows prominent subsarcolemmal accumulation of mitochondria in most fibers with ragged red-like fibers. (**B**) COX staining shows no COX-negative fibers, but there is marked subsarcolemmal staining. (**C**) SDH staining shows marked subsarcolemmal staining. (**D**) Oil red staining indicates moderate increase in lipid stores.

Patient 3 (F1-II-4) in Family 1 is the younger brother of patient 1 (F1-II-2), a 24-year-old male (only limited clinical data available). He has optic atrophy with ataxia and seizure disorder. An EGG was not performed. A left quadricep muscle biopsy was performed at the age of 10 years. A microscopic examination of the hematoxylin and eosin (H&E)-stained frozen sections revealed mild variation in myofiber size with no evidence of fibrosis, fatty replacement, or inflammatory infiltrate. Modified trichrome stain showed numerous myofibers with prominent subsarcolemmal fuchsinophilic material accumulation, with some ragged-red-like fibers. This material is mitochondrial in nature, as demonstrated on succinate dehydrogenase (SDH) and modified SDH reactions, and to a less extent on cytochrome *c* oxidase (COX) stain. Oil red-O showed a mild to moderate increase in neutral lipid stores. These results are shown in Figure 1A–D.

#### 3.1.4. Patient 4 (Family 2)

Patient 4 (F2-II-1) in Family 2 is a 25-year-old male (only limited clinical data available). He is the older brother of patient 5 (F2-II-2) and Patient 6 (F2-II-3). He is developmentally delayed and has a seizure disorder. EGGs have been performed many times at different ages, finding suggestive of mild-to-moderate cortical dysfunction and nonspecific encephalopathy predominantly in the left frontotemporal head region.

#### 3.1.5. Patient 5 (Family 2)

Patient 5 (F2-II-2) in Family 2 is a 16-year-old female born to healthy consanguineous parents. The pregnancy was full-term without difficulty. She is intellectually disabled and developmentally delayed in speech and motor skills. Nystagmus and seizure disorder were noted. An EGG that was performed at the age of 9 years old showed frequent spikes and sharp waves predominantly arising from the left frontotemporocentral head region and independently from the right frontotemporal, with intermittent slow activity, generalized, maximally in the left frontotemporocentral head region. This finding was suggestive of cortical irritability and epileptiform activity, which might predispose to focal seizures with and without secondary generalization. The presence of slow activity is indicative of cortical dysfunction and nonspecific encephalopathy.

#### 3.1.6. Patient 6 (Family 2)

Patient 6 (F2-II-3) in Family 2 is an 18-year-old male. This patient has a neurodevelopmental phenotype similar to his siblings.

### 3.2. Brain Imaging Findings

Patient 1 (F1-II-2) underwent magnetic resonance imaging (MRI) at the ages of 9 and 10 years old, showing evidence of optic atrophy and a cluster of flow void signal structures at the interhemispheric fissure anterior to the genu of the corpus callosum with larger pericallosal flow void structure worrisome for pial AVM (arteriovenous malformation).

Patient 2 (F1-II-1) underwent an MRI at the age of 15 years old and showed optic atrophy and an incidental small left frontal developmental venous anomaly and no evidence of abnormal enhancement. There was an unremarkable appearance and normal volume of the deep grey nuclei. The grey–white matter differentiation was maintained, and the major intracranial vascular flow voids were preserved. A smaller volume of the right hippocampus was questioned, as appreciated by the thick coronal acquired images, although with the possibly abnormal appearance of the left hippocampus, as suggested by a loss of intrinsic details, temporal lobe examination can be considered for specific mesial temporal structural assessment. The brain MRI of all other patients also showed optic atrophy.

**Figure 2:**
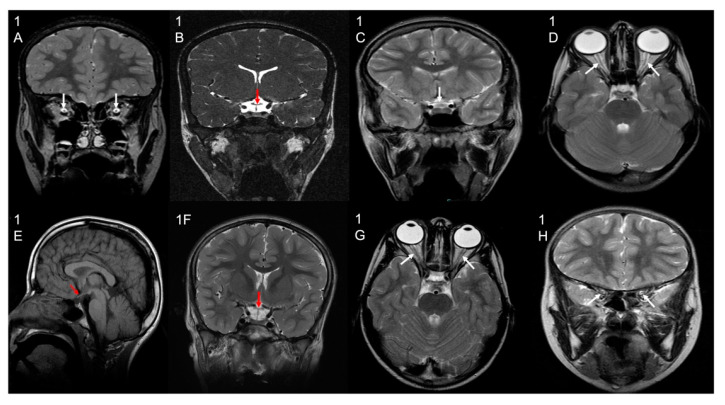
Brain MRI results. Selected MRI sequences and planes showing optic chiasm atrophy (white/red/black arrows) in patient 1 (image (**1B**) coronal CISS), Patient 2 (image (**1C**) coronal T2), Patient 4 (image (**1E**) sagittal T1), and Patient 5 (image (**1F**) coronal T2). Other selected images also show bilateral optic nerve atrophy (white arrows) in patient 1 (image (**1A**) coronal T2), Patient 3 (image (**1D**) axial T2), and Patient 6 (image (**1G**) axial T2; image (**1H**) coronal T2).

**Figure 3:**
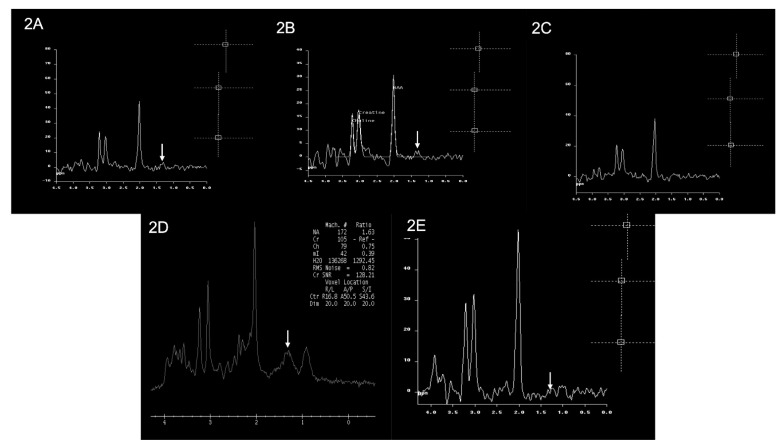
Brain MRS findings of the patients. Multiple single-voxel MRS studies showed small lactate doublets at 1.3 ppm (white arrows) in Patient 1 (image (**2A**)), Patient 3 (image (**2B**)), Patient 5 (image (**2D**)), and Patient 6 (image (**2E**)). There is no obvious lactate doublet seen in Patient 4 (image (**2C**)). An MRS study was not performed for Patient 2.

The MRS (magnetic resonance spectroscopy) for patients 1, 3, 5, and 6 showed a small lactate peak, and in patient 4, the peak was undetected, suggestive of metabolic disorder. Patient 2 did not have MRS performed. The MRI and MRS findings of the patients are shown in Figure 2 and Figure 3, respectively.

Clinical details of the patients are listed in Table 1.

### 3.3. Genetic and Molecular Results Evaluations

**Figure 4:**
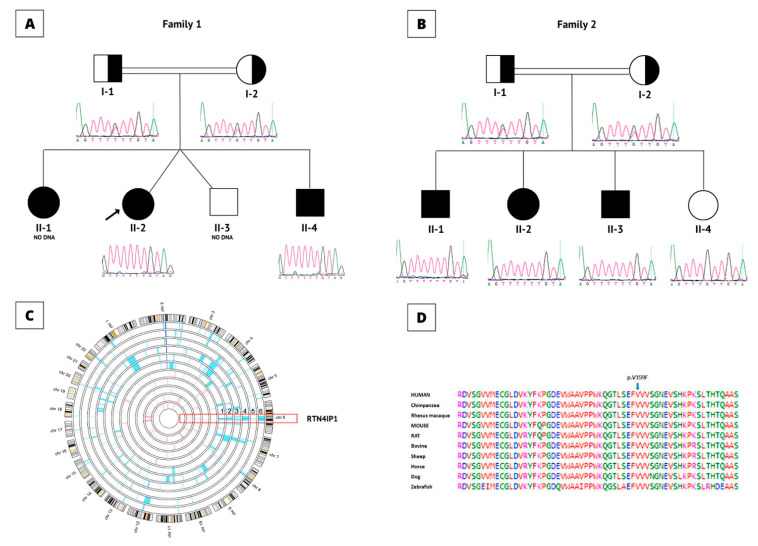
Genetic analysis results. (**A**) Pedigree of the Family 1. Affected individuals are labeled with black-colored (filled) symbols. Carriers are represented with half-filled symbols; squares represent males, circles represent females, and double line indicates consanguinity. (**B**) Pedigree for the family 2. Brief Sanger sequencing results of the c.475 G>T variant under the pedigrees show the results for the Families. (**C**) Homozygosity results for all the affected individuals in both families. The patients are aligned with the normal individuals in both families. The shared ROH block is shown in the red bracket on chromosome 6 where RTN4IP1 is located. Numbers 1-6 indicate family members from Family 1, individuals 1 to Family 2, and individual 3, (F1-II-1, F1-II-2, F1-II-3, F2-II-1, F2-II-2, and F2-II-3), respectively. (**D**) Protein sequence alignment of RTN4IP1, the blue arrow shows the affected Valine amino acids at position 159 by the novel variant. The alignment showed that the amino acid in this region is highly conserved among different species.

**Figure 5:**
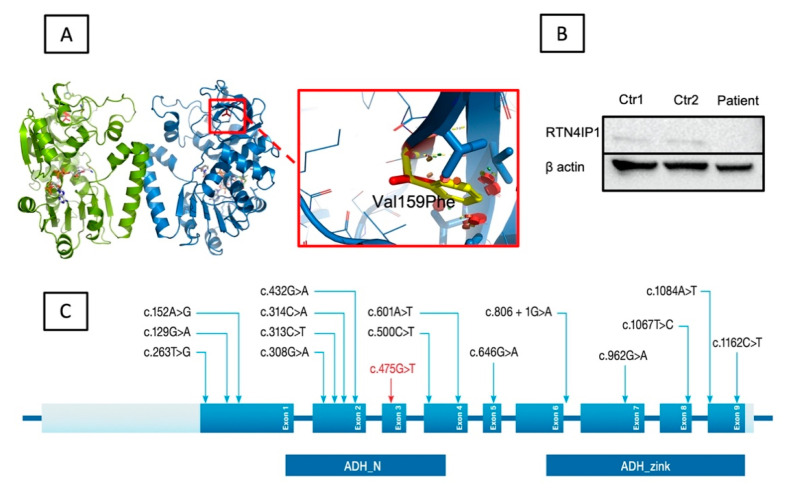
Structural analysis and immunoblotting experiment results. (**A**) The location of the variant within the 3-D crystal structure of RTN4IP1 (PDB ID: 2VN8). RTN4IP1 forms a dimer (green and blue chains). Val159 is shown as a red stick model. The cofactor NADPH is shown as a stick model with carbon atoms in white. The zoom view shows the effect of the p.Val159Phe substitution. Clashes of the Phenylalanine side chain (yellow) are shown as red discs. Key side chains in the vicinity are highlighted as stick models. (**B**) Immunoblotting analysis results. The Western blotting analysis revealed significantly decreased RTN4IP1 protein levels in the patient’s extracts from cultured fibroblasts in comparison to controls. A polyclonal antibody was targeted against the protein, and beta-actin was used as a control-loading marker. (**C**) Schematic presentation of *RTN4IP1*. The top panel shows localization of all previously reported variants (black) and the newly identified novel variant (red) distributed among the nine exons (blue). The bottom panel shows both the alcohol dehydrogenase domain (ADH_N) and the zinc-binding motif (ADH_Zinc).

In this study, we recruited families (Figure 4A,B) that showed increased lactic acidosis with an unknown cause and sought the possibility of finding novel nuclear genes. The initial sequencing of the whole mitochondrial genome failed to identify any pathogenic variants. Subsequently, we performed autozygosity mapping on the DNA collected from the members of the first family identified (Family 1) (Figure 4A). This analysis yielded a large “runs of homozygosity” (ROH) block on chromosome 6 specific to the affected individuals in Family 1. As a next step, we wanted to see if there was an additional family sharing the interval. This approach yielded a second family (Figure 4B) with a similar ROH. This ROH was confirmed when the available family members (affected individuals vs. normal individuals) were interrogated for the shared autozygome. All of the patients (n = 6) had a common ROH aligned on chromosome 6 (Figure 4C). We then inquired whether the clinical details of the patients from the second family shared similarities to those of the first family. This indeed revealed significant overlapping of the clinical manifestations between the affected individuals in both families. Next, two patients (F1-II-2 and F2-II-1) from these families were analyzed using WES. Using previously published approaches [17,18,20,28,29,30], our analysis revealed the same variant of *RTN4IP1* (NM_032730.5:exon3:c.475G>T, p.Val159Phe) as being the most probable target for further study. Both family members were interviewed separately and stated that they are unrelated and have no known close familial relationship. However, we learned that, although both families are unrelated, they indeed share the same tribal kinship. We utilized Sanger sequencing to determine the segregation of the variant in both families. This analysis revealed complete segregation of the variant in both families (Figure 4A,B). We also checked the presence of the variant in local and international databases, including commercially available ones. Based on the Human Gene Mutation Database (HGMD) [31] and others, the variant was novel and not present among more than 2500 in-house exomes, including our in-house exomes and local databases such as Saudi Human Genome Database (SHGP) and King Abdullah International Medical Research Center (KAIMRC) Genomic Database (KGD). The variant’s frequency (homozygous) was found to be 0.000420344682639765, and none (as heterozygous) was found in the searched databases. An alignment of the amino acid sequences surrounding the region of the variant site across different species showed that valin is highly conserved in position 159, suggesting the functional importance of this residue (Figure 4D). The crystal structure of RTN4IP1 (PDB ID: 2VN8) shows that Val159 is part of the ADH-N domain. Val159 is buried inside the hydrophobic core of this domain and approximately 20Å away from the catalytic site (Figure 5A). The replacement of the symmetric small, aliphatic valine side chain by larger aromatic phenylalanine is predicted to result in marked steric clashes in one direction but leave a gap in the other direction. Therefore, Val159Phe is expected to severely destabilize the ADH-N domain and hence render the whole protein unstable and prone to degradation. In agreement, the substitution is predicted to be deleterious by SIFT [22], Polyphen-2 [23], SNPdryad [27], MutationTaster [24,25], and Condel [26].

To investigate the potential consequence of the missense variant on protein expression, we performed immunoblotting experiments using the index patient’s (F1-II-2) fibroblasts. Our results revealed a marked loss of RTN4IP1 steady-state levels in the fibroblasts of the patient as compared to those of the two controls (Figure 5B). This result is consistent with previous findings, which showed a complete loss of RTN4IP1 protein in fibroblasts and muscle biopsy samples from the patients harboring variants in *RTN4IP1* accompanied by a reduction in complex I protein levels and mitochondrial complex I assembly [10].

Since the families belong to the same tribe, we hypothesized the presence of a founder effect. Therefore, we performed an autozygosity scan to identify ROH blocks throughout the genome. The analysis indicated a shared block (specific to the patients) on chromosome 6 in which RTN4IP1 is located. We utilized this ROH that spans ~3.6 centimorgans (cM) based on deCODE Map (GRCh37/hg19 Assembly). Using this, we calculated the recombination rates to calculate a predicted age for the variant. The calculation was performed using published protocols [1,19,32]. This analysis predicted that the variant could be traced back 56 generations. Assuming a mean generation of 25 years, the variant is estimated to have appeared approximately 1400 years ago.

## 4. Discussion

Although *RTN4IP1* is reported to encode a mitochondrial protein interacting with UQCRC1 and UQCRC2 within the mitochondrial respiratory chain, recent studies have linked its function to Complex I and CoQ biosynthesis [5,9]. According to Ensembl [33], *RTN4IP1* has two main transcripts and two additional processed splice variants that do not code for any protein. The two main transcripts encode 396 (UNIPROT:Q8WWV3; ENSP00000358059.3) and 226 (UNIPROT: G3V1R2; ENSP00000444261.1) amino acids, respectively. The expression of the gene is ubiquitous in various human tissues, especially in the central nervous system as well as in mitochondria-enriched tissues. For instance, screening a panel of human tissues revealed moderate expression in the brain, liver, and placenta, while high expression was present in the skeletal muscle, the kidney, and the heart [5]. A significant immunoreactivity was observed in the neuronal cells and astrocytes [5]. Hence, during the discovery of the first hereditary human variants, the patients were tested for mitochondrial deficiencies in oxidative phosphorylation, which have been found to primarily lead to complex I and IV deficiencies in patient fibroblasts [3,10]. In a follow-up study, muscle biopsies were also analyzed, and an isolated complex I deficiency was identified in the patient’s muscles, with no evidence of complex IV deficiency [10]. The clinical consequences of these variants have been associated with early-onset optic neuropathy [3,12], optic atrophy [10], rod-cone dystrophy [11], and severe encephalopathy with optic atrophy [10].

To date, several mutations have been identified in different patients, including 15 single-nucleotide change mutations and two deletion mutations) in three separate studies [3,4,10,11,12,13,14,34,35], as summarized in Table S1 and shown in Figure 5C. Thereof, 15 variants cause various single amino acid changes, two variants lead to truncations, and one variant presumably causes aberrant splicing. Interestingly, the splice site variant was reported in two studies as a compound heterozygous mutation [10,12] and was found to be a de novo mutation [4,10,12,14].

The effect of *RTN4IP1* variants on RTN4IP1 function may range from mild to severe depending on the type of variant being nonsense or splice site. Optic atrophy, seizures, nystagmus, and global developmental delay are the most common symptoms associated with pathogenic variants in the *RTN4IP1*. In addition, deafness, brain MRI abnormalities, stridor, and abnormal electroencephalograms were seen in severely affected patients with premature death due to the deleterious effect of the variants on RTN4IP1 activity and structure [9,13].

Our index patient (F1-II-2) had been severely affected by epileptic seizures from early childhood. She is now free from seizures, and there has been no further progression of the disorder. Our patients showed low visual acuity. Their ocular size was normal with orbital hypotelorism. Our results further support the suggestion that *RTN4IP1* abnormalities result in early-onset optic neuropathy and neurological features, including mild intellectual disability and epilepsy. The clinical manifestations resemble mitochondrial encephalopathy.

The patients with the identified variant in *RTN4IP1* belong to unrelated consanguineous Saudi families. All of the previously identified variants in *RTN4IP1*, as well as our novel variant (c.475G>T, p.Val159Phe), are shown in Figure 5C. The variant was predicted to be pathogenic based on various in silico classifiers and structural modeling. As shown in Figure 5A, the variant sits in a functionally critical domain, which is one of the two domains of the protein with quinone oxidoreductase activity [10]. The substituted valine is highly conserved (Figure 4D), and part of the hydrophobic core of the domain (Figure 5A) is close to the catalytic activity site of the protein. Such a replacement, from a small valine molecule to a much larger phenylalanine, will introduce marked unidirectional steric clashes likely to cause a directional gap that leads to severe destabilization in the domain. Such an effect will result in an unstable protein that is prone to degradation. Relatedly, our immunoblotting experiments revealed the absence of the protein in the index patient’s fibroblasts (patient1, F1-II-2) (Figure 5B). Hence, this suggests that the variant affects a functionally important residue.

Since the families belong to a specific tribe, we assumed the likely involvement of a founder effect. To calculate the age of the founder variant, we analyzed genome-wide autozygosity scan results that revealed a patient-shared ROH inclusive of *RTN4IP1* on chromosome 6. The analysis predicted that the variant passed 56 generations, first appearing 1400 years ago (assuming a mean generation of 25 years). The presence of such founders is occasionally encountered in our population.

In conclusion, we report a novel deleterious variant in *RTN4IP1* leading to intellectual disability, encephalopathy, ataxia, optic atrophy, and seizures in our patients. To our knowledge, this is the first founder variant of the gene. The identification of population-specific pathogenic variants will help faster diagnosis and precise genetic testing in consanguineous populations.

## Figures and Tables

**Table 1:** Clinical features of the reported patients in this study.

Family	Family 1			Family 2		
Patient Number	Patient 1	Patient 2	Patient 3	Patient 4	Patient 5	Patient 6
Patient Codes	F1-II-1	F1-II-2	F1-II-3	F2-II-1	F2-II-2	F2-II-3
Gender	Male	Female	Male	Male	Female	Male
Age (years)	29	27	25	26	17	19
Parental Consanguinity	Yes	Yes	Yes	Yes	Yes	Yes
Age of onset (years)	Not available	Not available	Not available	2	1.5	1.5
Microcephaly	No	No	No	No	No	No
Developmental delay	Yes	Yes	Yes	Yes	Yes	Yes
Axial Hypertonia	No	No	No	No	No	No
Axial Hypotonia	No	No	No	No	No	No
Other movement disorder	Unknown	Unknown	Unknown	Unbalanced and delayed walking	Lost ability to walk	Unbalanced and delayed walking
Optalmological findings	Nystagmus, optic atrophy	Nystagmus, optic atrophy	Nystagmus, optic atrophy	Optic atrophy	Optic atrophy	Optic atrophy
Seizure	Generalized tonic clonic	Generalized tonic clonic	Generalized tonic clonic	Generalized tonic clonic	Generalized tonic clonic	Generalized tonic clonic
White matter abnormalities	No	No	No	No	No	No
Left hemispheres abnormalities	No	No	No	No	No	No
Cerebral encephalopathy	Yes	Yes	Yes	Yes	Yes	Yes
Temporal areas abnormalities	No	No	No	No	No	No
Subdural area abnormalities	No	No	No	No	No	No
Levetiracetam	No	No	No	No	No	No
Phenytoin	No	No	No	No	No	No
Carbamazepine	No	Yes	No	No	Yes	No
Phenobarbital	No	No	No	No	No	No
Clonazepam	No	No	No	No	No	No
Topiramate	No	No	No	No	No	No
NCBI Reference ID	NM:032730	NM:032730;	NM:032730	NM:032730	NM:032730	NM:032730
cDNA Change	c.475G>T	c.475G>T	c.475G>T	c.475G>T	c.475G>T	c.475G>T
Amino Acid Changes	p.Val159Phe	p.Val159Phe	p.Val159Phe	p.Val159Phe	p.Val159Phe	p.Val159Phe
Others	-	-	-	-	Bulbous nose, curved relatively small ears, hypertelorism, synopsis large upper lip, short philtrum and skin eczema in limbs	-

## Data Availability

Not applicable.

## Supplementary Materials

The following supporting information can be downloaded at: https://www.mdpi.com/article/10.3390/cells11193154/s1, Table S1: Summary of the clinical and genetic findings for patients with *RTN4IP1* variants.
