# Supplementary material for: A Novel Homozygous Founder Variant of RTN4IP1 in Two Consanguineous Saudi Families
Source: Cells. 2022 Oct 7;11(19):3154. doi: 10.3390/cells11193154 (PMC9563936; doi:10.3390/cells11193154)
Supplement: Supplementary file 1 [file cells-11-03154-s001.zip › cells-1898025-supplementary.pdf]

Supplementary Table S1. Summary of the clinical and genetic findings for patients with *RTN4IP1* mutations (The variants are ordered according to the base numbering on the cDNA)

Patient	Mutation	Protein	Respiratory chain activity	Epilepsy	Brain MRI	Developmental Delay	Other symptoms	References
Case study	c.307C>T, deletion	p.Arg103Cys, deletion	Unknown	No	Unknown	No	No	*Jurkute et al., 2022*
Family I, II-3, F	c.308G>A	p.Arg103His	CI and CIV deficits	No	Unknown	No	No	*Angebault et al., 2015*
Family I, II-4, M	c.308G>A	p.Arg103His	CI and CIV deficits	No	Unknown	No	No	*Angebault et al., 2015*
Family II, II-1, M	c.308G>A	p.Arg103His	Unknown	No	Normal	No	No	*Angebault et al., 2015*
Family III, II-5, F	c.308G>A	p.Arg103His	Unknown	No	Unknown	No	No	*Angebault et al., 2015*
Family IV, II-2, F	c.308G>A; c.601A>T	p.Arg103His; p.Lys201*	CI and CIV deficits	Yes	Thin optic tracts	Mild	Nystagmus, mild ataxia, learning disabilities	*Angebault et al., 2015*
Family IV, II-3, F	c.308G>A; c.601A>T	p.Arg103His; p.Lys201*	CI and CIV deficits	No	Thin optic tracts	Mild	Nystagmus, mild ataxia, learning disabilities	*Angebault et al., 2015*
II-1, M	c.308G>A; c.806+1G>A	p.Arg103His; splicing	Unknown	Yes	Hypoplastic optic nerves	No	Nystagmus, learning difficulty, transient hyperlacticacidemia	*Okamoto et al., 2017*
II-2, M	c.308G>A; c.806+1G>A	p.Arg103His; splicing	Unknown	No	Hypoplastic optic nerves	Mild	Nystagmus	*Okamoto et al., 2017*
Family I, II-1	c.308G>A	p.Arg103His	Unknown	No	Normal	No	Nystagmus	*Charif et al., 2017*
Family 2, II-1	c.308G>A	p.Arg103His	Unknown	No	Normal	No	No	*Charif et al., 2017*
Family 2, II-4	c.308G>A	p.Arg103His	Unknown	No	Normal	No	No	*Charif et al., 2017*
Family 3, II-1	c.308G>A	p.Arg103His	Unknown	No	Normal	No	No	*Charif et al., 2017*
Family 4, II-1	c.308G>A	p.Arg103His	Unknown	No	Unknown	No	No	*Charif et al., 2017*
Case study	c.308G>A	p.Arg103His	Unknown	No	Isolated small subependymal heterotopia and asynchronous self-resolving midbrain lesions.	No	Nystagmus, myopia, mild intellectual disability, generalized chorea,	*Giacomini et al., 2020*
Family 1, II-3&II-4	c.308G>A	p.Arg103His	Unknown	No	Unknown	No	No	*Meunier et al., 2021*
Family 2,II-5	c.308G>A	p.Arg103His	Unknown	No	Unknown	No	No	*Meunier et al., 2021*

Family 3,II-4&II-5,III-2	c.308G>A	p.Arg103His	Unknown	No	Unknown	No	Nystagmus	*Meunier et al., 2021*
Family 4,II-2&II-3	c.308G>A; c.601A > T	p.Arg103His; p.K201*	Unknown	No	Unknown	No	No	*Meunier et al., 2021*
Case study	c.432G>A, c.646 G>A	p.W144X, p.G216R	Unknown	No	Normal	No	No	*Rajabian et al., 2021*
Family 5, II-1	c.1084A>T	p.Ile362Phe	Unknown	No	Unknown	No	No	*Charif et al., 2017*
Family 6, II-4	c.129G>A; c.152A>G	p.Trp51Cys; p.Met43Ile	Unknown	No	Normal	No	No	*Charif et al., 2017*
Family 7, II-2	c.313C>T	p.Pro105Ser	Unknown	Yes	Normal	Severe	Elevated plasma lactate, poor feeding, nonverbal	*Charif et al., 2017*
Family 8, II-1	c.1067T>C	p.Leu356Pro	Unknown	Yes	Unknown	Yes	Hypotonia at birth, nystagmus, ataxia, lower extremity spasticity	*Charif et al., 2017*
Family 9, II-2	c.432G>A; c.962G>A	p.Trp144*; p.Gly321Glu	CI deficit	Yes	T2 hyperintensities in thalami, subthalamic and dentate nuclei, and brainstem	Yes	Elevated serum lactate and pyruvate, spastic tetraparesis, upper extremity dystonia, abnormal AEPs	*Charif et al., 2017*
Family 10, II-1	c.314C>A	p.Pro105His	CI deficit	Yes	Cerebral atrophy	Yes	Hypotonia, stridor	*Charif et al., 2017*
Family 11, II-2	c.500C>T; c.806+1G>A	p.Ser167Phe; splicing	CI deficit	Unknown	T2 high signal change and swelling in posterolateral putamina		Elevated plasma lactate and alanine, poor feeding, tracheostomy	*Charif et al., 2017*
II-1, F	c.646G>A; c.1162C>T	p.Gly216Arg; p.Arg388*	Unknown	Yes	Normal	Mild	Nystagmus, elevated serum lactate and pyruvate	*Zou et al., 2019*
II-2, M	c.646G>A; c.1162C>T	p.Gly216Arg; p.Arg388*	Unknown	Yes	Normal	Mild	Nystagmus, elevated serum lactate and pyruvate	*Zou et al., 2019*
II-1, M	c.263T>G; deletion	p.Val88Gly; deletion	Unknown	Yes	Pituitary cysts, T2 hyperintensities	Yes	Nystagmus, ataxia, choreoathetosis, hypotonia, elevated plasma lactate	*Present study*
Case study	c.263T>G	p.Val88Gly	Unknown	Yes	T2 signal abnormalities	Yes	Nystagmus, ataxia, and Choreoathetosis	*D'Gama et al., 2021*

GenBank: NM_032730; CI: Complex I, CIV: Complex IV, AEPs: auditory evoked potentials
